# Bioactive Glass Fiber-Reinforced PGS Matrix Composites for Cartilage Regeneration

**DOI:** 10.3390/ma10010083

**Published:** 2017-01-20

**Authors:** Marina Trevelin Souza, Samira Tansaz, Edgar Dutra Zanotto, Aldo R. Boccaccini

**Affiliations:** 1 CeRTEV—Center for Research, Technology and Education in Vitreous Materials, Vitreous Material Laboratory, Department of Materials Engineering, Universidade Federal de São Carlos—UFSCar, 13565905 São Carlos, SP, Brazil; dedz@ufscar.br; 2 Institute of Biomaterials, University of Erlangen-Nuremberg, 91058 Erlangen, Germany; samira.tansaz@fau.de (S.T.); aldo.boccaccini@ww.uni-erlangen.de (A.R.B.)

**Keywords:** poly(glycerol sebacate), bioactive glass, fibers, composite, cartilage, tissue engineering

## Abstract

Poly(glycerol sebacate) (PGS) is an elastomeric polymer which is attracting increasing interest for biomedical applications, including cartilage regeneration. However, its limited mechanical properties and possible negative effects of its degradation byproducts restrict PGS for in vivo application. In this study, a novel PGS–bioactive glass fiber (F18)-reinforced composite was developed and characterized. PGS-based reinforced scaffolds were fabricated via salt leaching and characterized regarding their mechanical properties, degradation, and bioactivity in contact with simulated body fluid. Results indicated that the incorporation of silicate-based bioactive glass fibers could double the composite tensile strength, tailor the polymer degradability, and improve the scaffold bioactivity.

## 1. Introduction

Cartilage is an avascular, aneural, and low-metabolic-activity tissue that presents very limited regenerative potential [1,2]. Therefore, any defect, deterioration, or damage caused by trauma, disease, or aging is a limiting condition for the patient, impairing their normal life [1,2].

Damage in cartilage is normally managed by the use of analgesics and physical therapy; however, in severe cases, surgery is required, which leads to immediate to long-term complications [1]. Developing a biomaterial that can sustain cell growth with suitable mechanical properties for cartilage regeneration is an important challenge in tissue engineering.

Regarding these characteristics, poly(glycerol sebacate) (PGS) is a biocompatible, biodegradable, elastomeric polymer, which has shown great potential as a scaffold material for soft and hard tissue engineering applications [3].

PGS is a synthetic polymer, firstly reported in the context of tissue engineering in 2002 [4]. This elastomer is relatively inexpensive, exhibits thermoset elastomeric properties, and its in vivo degradation products can be eliminated through natural pathways [3]. PGS has been already applied in several studies with satisfying results regarding the regeneration of cardiac muscle [5,6,7], vascular tissue [8,9], cartilage [10,11], nerve conduits [12,13], retina [14,15], and tympanic membrane perforations [16,17].

In any tissue engineering strategy, it is important to tailor the rate of degradation of the biomaterial to match the regenerative rate of the engineered tissue. PGS has been reported to undergo fast degradation in vivo, being completely absorbed within 6 weeks [4]. It has been demonstrated that by incorporating bioactive glass 45S5 in PGS [18] and by coating bioactive glass scaffolds with PGS, the degradation rate of the composites can be tailored to attenuate the composites’ degradation kinetics to match that of the targeted tissues. The combination of bioactive glass with PGS can also potentially overcome possible problems associated with the toxicity presented by the acidic degradation products of PGS [3].

Recently, biocomposites reinforced with glass fibers have drawn considerable scientific attention due to the positive results achieved in terms of mechanical strength [19]. However, inert fibers are normally incorporated as a reinforcement agent, not contributing to the bioactivity of the biocomposite. Therefore, the manufacture of PGS-based biocomposites using bioactive glass fibers (BGF) would bring numerous advantages to this biomaterial, leading to higher mechanical properties and imparting bioactivity. For this purpose, the goal of this study was to develop a new PGS-based scaffold reinforced with bioactive glass microfibers for a potential use in cartilage regeneration.

## 2. Results

### 2.1. Biocomposite Structure

**Figure 1:**
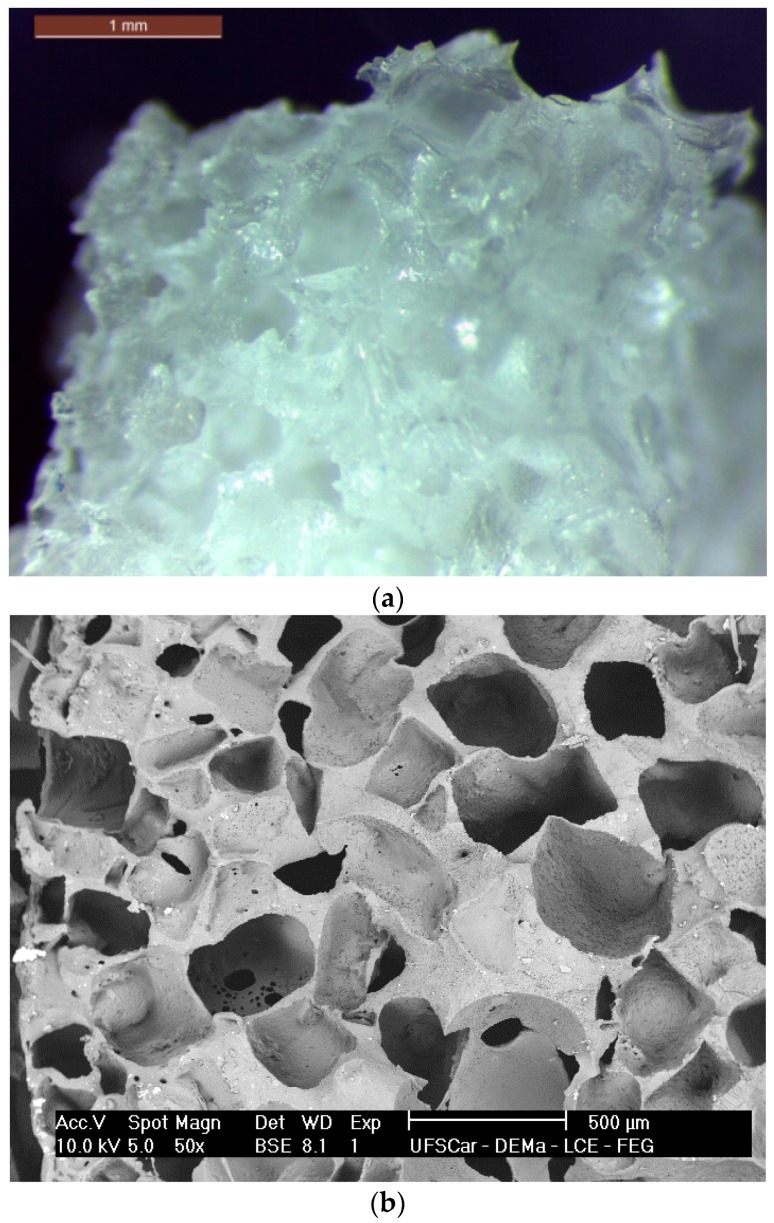
Morphology of the poly(glycerol sebacate) (PGS) scaffolds obtained via salt leaching. (**a**) Stereomicroscope image (scale bar: 1 mm) and (**b**) SEM analysis showing the porous structure.

Figure 1a,b present a general overview of the scaffolds morphology.

The obtained scaffolds had a medium pore size of 475 µm (±100 µm) with few pores reaching up to 1 mm. It is noticeable that a highly porous structure was developed. Based on image analysis (ImageJ, National Institutes of Health, Bethesda, MD, USA), the obtained scaffolds reached a mean porosity not lower than 60 vol %, with reasonable pore interconnectivity despite the used technique (salt-leaching process). The presence of the fibers is not perceptible since they are totally embedded in the polymer matrix, indeed acting as a reinforcement agent in the scaffold structure.

### 2.2. Degradation Tests

**Figure 2:**
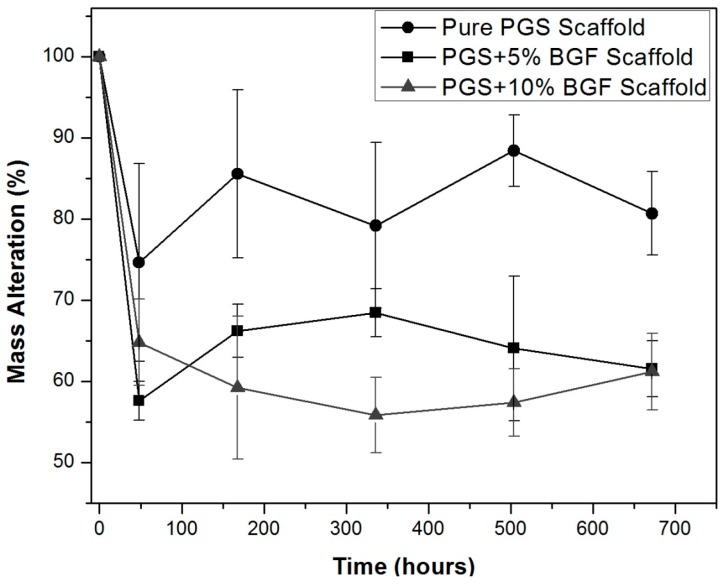
Weight loss (%) of pure PGS scaffolds and PGS + bioactive glass fibers (BGF) composite scaffolds (5% and 10%) in ISO degradation solution over time.

Figure 2 presents the porous composites’ degradation and weight loss over time when soaked in the degradation solution proposed by the ISO 10993 standard [20]. For all samples, a weight loss was observed, mainly after 48 h, and presented no significant weight change over longer incubation periods. As expected, a greater weight loss was presented by the composite scaffolds in comparison to the PGS matrix because of the rapid dissolution of the glass fibers that were exposed to the medium. Due to the similarities and the great standard deviation found for these samples, no statistical difference between them could be detected when a *t*-test was applied.

**Figure 3:**
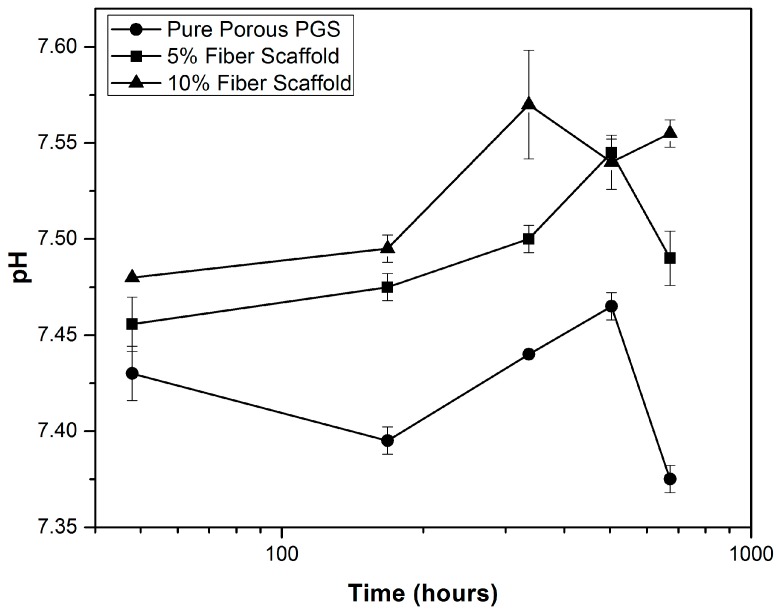
pH variation over time in ISO degradation solution for PGS scaffolds with and without bioactive fiber reinforcement.

The variation of pH in the ISO degradation solution over time can be observed in Figure 3. For the pure PGS scaffolds, a slight decrease in pH was observed, reaching a minimum value of 7.37 at day 28. However, for the PGS + BGF composites, an increase in pH was observed, with higher values reached for the sample with the greater fiber content (7.57 at day 14 for the PGS + 10% BGF scaffold).

**Figure 4:**
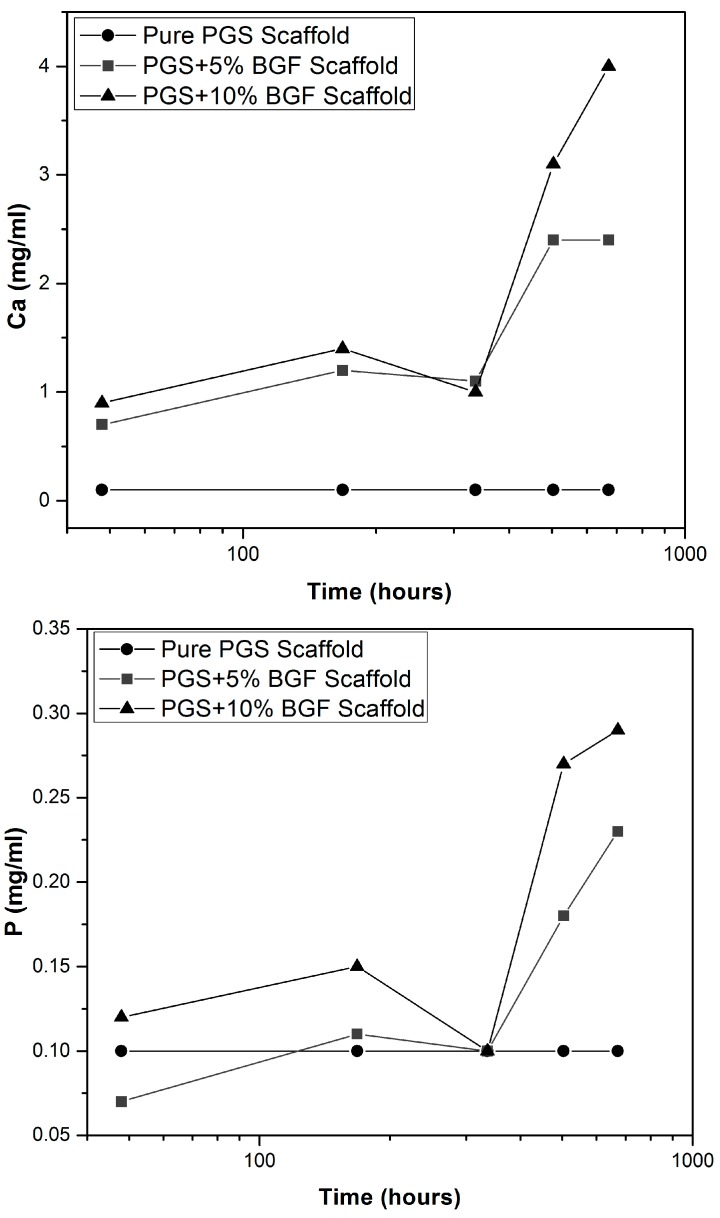

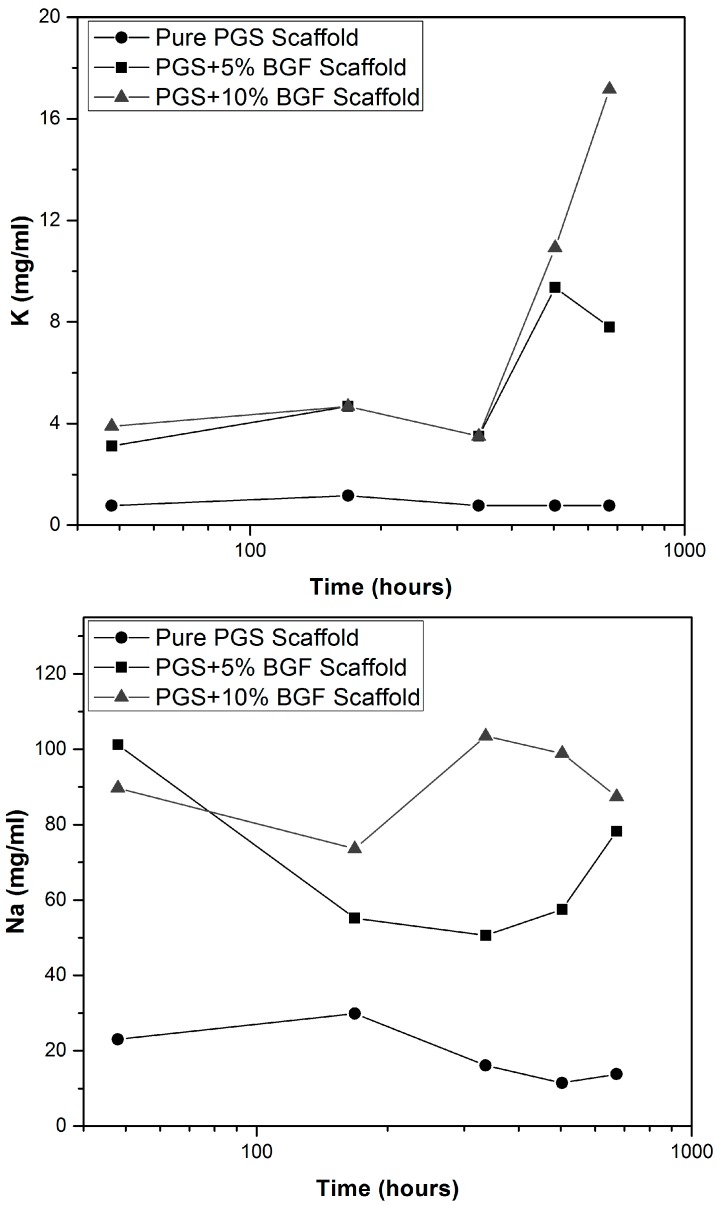
Ion release vs. time in ISO degradation solution for pure PGS scaffolds and PGS + BGF samples with 5% and 10% of the reinforcement agent.

Ion-selective tests (Figure 4) showed that the amount of Ca, P, K, and Na released into the ISO degradation solution was kept constant for the pure PGS scaffolds for all experimental periods. Yet, for both PGS + BGF samples (5% and 10%), an increase in the amount of Ca, P, Na and K was detected. The quantity of ions reached a maximum value mainly after 28 days, which was higher for PGS + 10% BGF samples, as expected, due to the greater amount of the bioactive phase. This ion release burst linked to the dissolution of the bioactive glass is well known and has been already reported in the literature [21].

### 2.3. Bioactivity Tests

**Figure 5:**
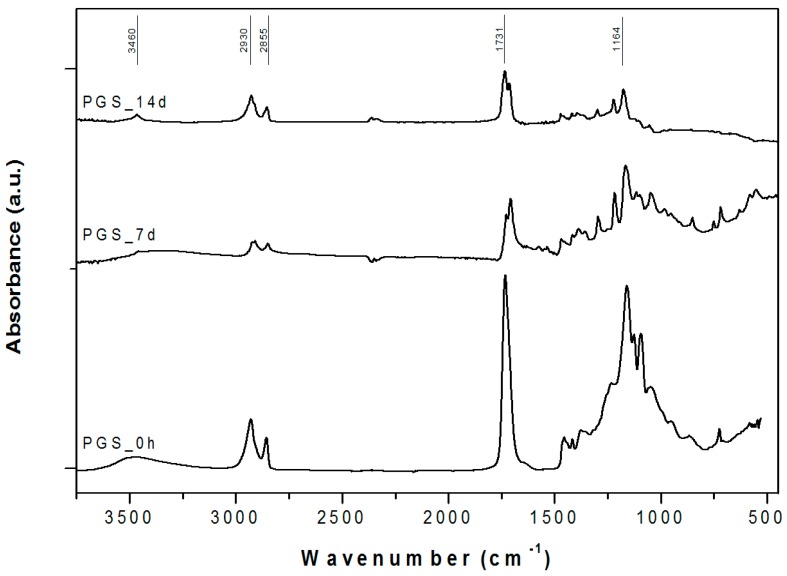
ATR-FTIR spectra for pure PGS before and after simulated body fluid (SBF) in vitro tests for 7 and 14 days. The relevant peaks are discussed in the text.

Attenuated total reflectance Fourier-transform infrared spectroscopy (ATR-FTIR) confirmed the formation of ester bonds in PGS (Figure 5), as revealed by the intense peak at ≈1730 cm^−1^ (C=O stretch) and 1164 cm^−1^ (C–O) [18,22].

PGS samples also exhibited the two characteristic alkane groups (–CH_2_) absorption peaks at around 2925 cm^−1^ and 2855 cm^−1^ [22] and the broad peak around 3460 cm^−1^, linked to hydrogen-bonded hydroxyl groups [18].

As the soaking time in simulated body fluid (SBF) solution increased, the intensity of these PGS characteristic peaks decreased. This is an expected phenomenon linked to PGS degradation and hydrolysis, a trend reported in the literature [23,24].

**Figure 6:**
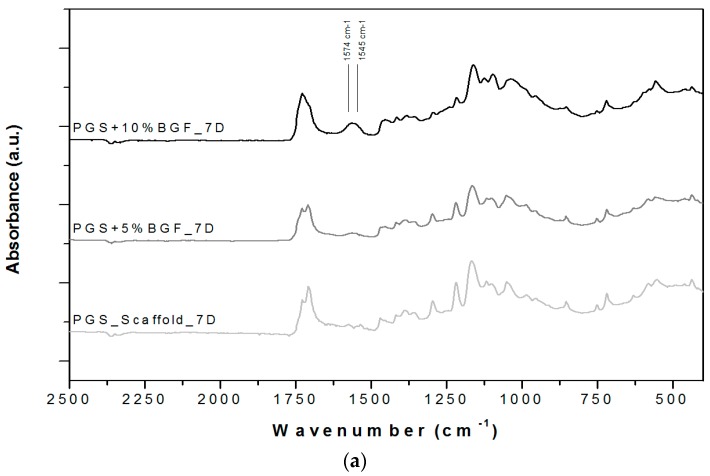

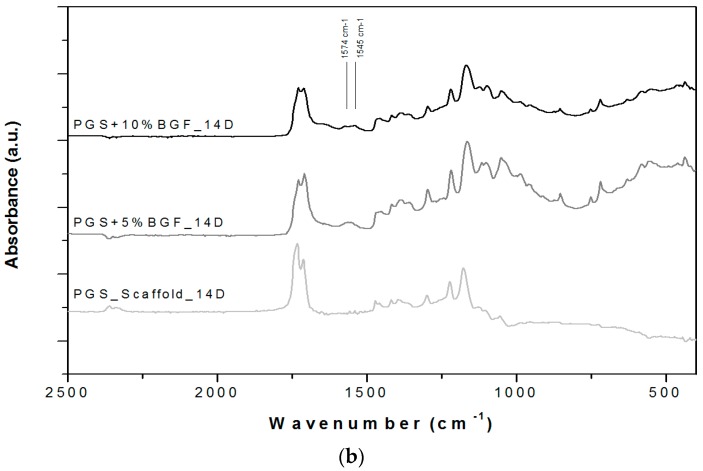
ATR-FTIR spectra for PGS scaffolds and PGS + BGF composites (**a**) after 7 days soaking in SBF solution and (**b**) after 14 days soaking in SBF solution. The relevant peaks are discussed in the text.

**Figure 7:**
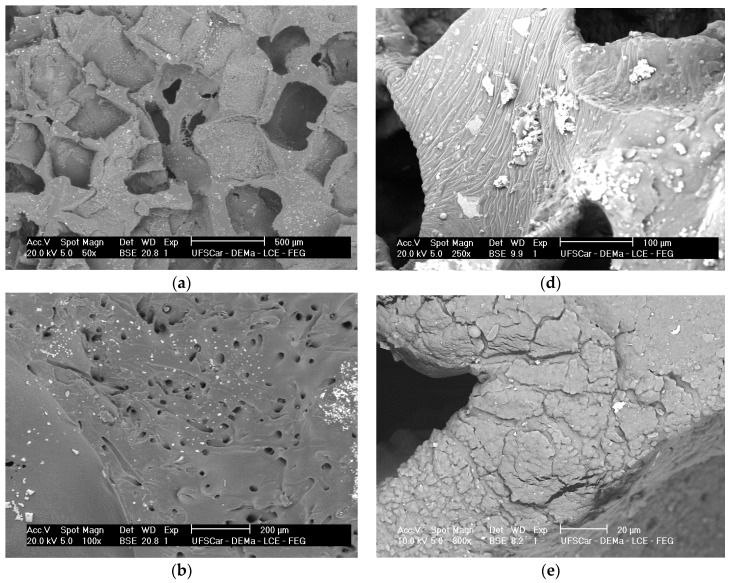

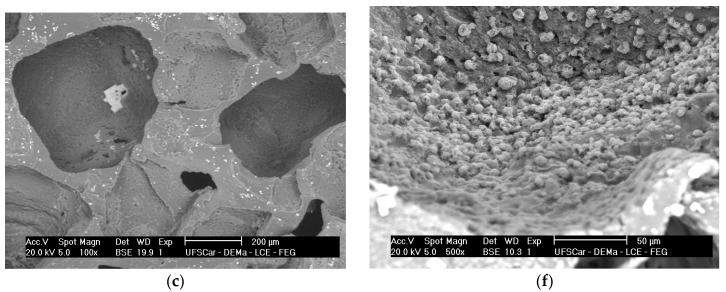
SEM images of (**a**) pure PGS scaffolds; (**b**) PGS + 5% fiber scaffold; and (**c**) PGS + 10% fiber scaffold soaked in SBF solution for 2 days; and (**d**) pure PGS scaffolds; (**e**) PGS + 5% fiber scaffold, and (**f**) PGS + 10% fiber scaffold soaked in SBF solution for 14 days.

ATR-FTIR spectra for the PGS + BGF scaffolds are presented in Figure 6. In all spectra (Figure 6a,b), it is possible to notice the presence of two new peaks with the addition of the bioactive glass fibers to PGS, at 1545 and 1574 cm^−1^. These peaks are more evident for the PGS + 10% BGF due to the greater amount of bioactive glass. For both composite samples, it is noticeable that these peaks lose intensity as the time of experimentation increased. In Figure 6, it is also possible to observe that the split of the peak at ≈1730 cm^−1^, with the appearance of the peak at 1700 cm^−1^—which is linked to PGS degradation—takes longer periods to occur when the bioactive glass fibers are added and are less intense, mainly for the PGS + 10% fibers samples. In these FTIR spectra, the formation of the hydroxycarbonate apatite (HCA) layer is not clearly detected; to verify the formation of this bioactive phase on the surface of the porous scaffolds, SEM images were taken. Figure 7 presents the globular structure linked to HCA inside the porous composites and on the surface of the composites after 2 and 14 days of soaking in SBF solution for (1) PGS + 5% BGF and (2) PGS + 10% BGF scaffolds. For pure PGS scaffolds, indication of polymer surface erosion is visible, which is likely the result of the hydrolysis degradation over time.

As can be observed, after 2 days in SBF, the HCA layer cannot be easily detected, but after 14 days of incubation this phase precipitated on both fiber concentrations biocomposite scaffolds.

**Figure 8:**
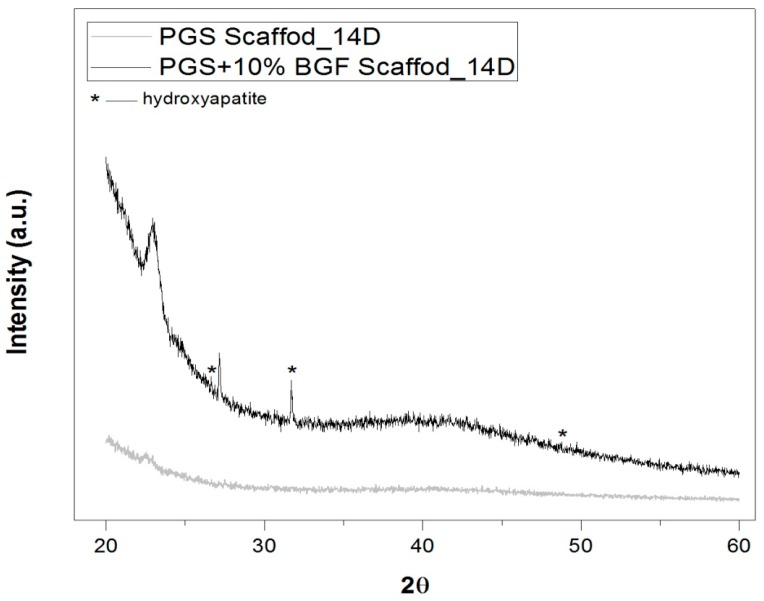
XRD spectra of the scaffolds (pure PGS scaffold and PGS + 10% BGF composites) after immersion in SBF solution for 14 days. The peaks of hydroxyapatite are marked by *. The found peaks were in good agreement with HA JCPDS card (09-0432).

Figure 8 presents the XRD pattern for the (1) pure PGS and (2) PGS + 10% BGF composite samples after 14 days of incubation in SBF solution. Although mainly amorphous material is detected (broad band at ≈22°), the confirmation of the formation of hydroxyapatite is possible, by considering the peaks at ≈26°, 32°, and 49° [25,26].

### 2.4. Mechanical Properties

**Figure 9:**
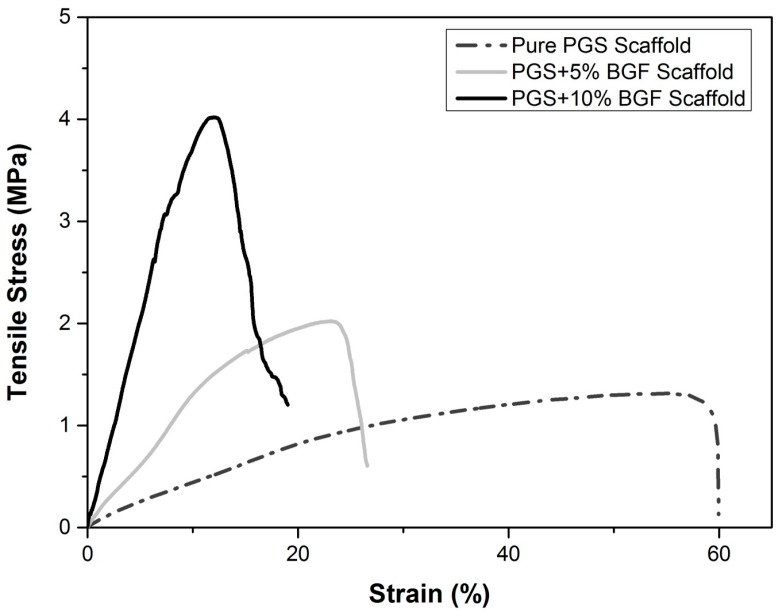
Typical curves of tensile stress for the pure porous PGS, PGS + 5% BGF, and PGS + 10% BGF scaffolds.

Typical tensile stress–strain curves for pure PGS porous scaffolds and for PGS + 5% and PGS + 10% BGF composite scaffolds are shown in Figure 9. The mean values for the maximum tensile strength and the maximum deformation, as well as their standard deviations, are presented in Table 1. The maximal tensile strength achieved for the pure PGS scaffolds was 1.2 MPa with a maximal elongation of 60%, an expected result given the relatively high porosity of the scaffolds. With the addition of the fibers into the porous structure, the composites showed a maximal tensile strength of approximately 2 and 4 MPa with a strain of 26% and 20% for PGS + 5% BGF and PGS + 10% BGF, respectively.

A *t*-test showed that the *p* value was <0.05 when the porous PGS group was compared to all other groups. On the other hand, between PGS + 5% and PGS + 10%, no significant difference was observed.

## 3. Discussion

Tissue engineering presents an alternative approach to repair and regenerate damaged tissues, eliminating the need for permanent implants. Thus, it is highly promising to tackle numerous medical needs. However, many challenges remain, and the pursuit of new, responsive, and appropriate biomaterials is continually growing [27].

Over the past years, numerous tissue-engineering strategies have been considered for cartilage tissue regeneration, using both naturally occurring and artificial polymeric biomaterials. Several studies have shown the potential of PGS in soft tissue regeneration [5,6,7,8,9] and, more specifically, cartilage regeneration [10,11].

PGS is a biocompatible, biodegradable, and mechanically stable elastomeric biopolymer that can be synthetized by many different routes, aiming to tailor its mechanical properties to match those of cartilage tissue [10]. As a highly porous scaffold, this polymer has demonstrated its capability to produce a cartilaginous matrix due to a higher chondrogenic gene expression when compared to poly(ɛ-caprolactone) (PCL) [3,10].

Aiming to achieve a more bioactive and mechanically suitable biomaterial for tissue regeneration, several researchers incorporated bioactive glass into PGS [3,18,26,28]. In this study, a new bioactive glass fiber was added to a porous PGS matrix, aiming at reinforcing the 3D scaffold structure and controlling the polymer degradation, counteracting the acidity of the PGS leachates.

The developed scaffolds exhibited a well-developed porosity obtained by the salt-leaching technique, with pore sizes spanning over a very wide range (from few microns to hundreds of microns), with no sign of agglomeration of the glass fibers. Regarding PGS degradation, it is currently well established that this polymer undergoes surface degradation, the main mechanism being the cleavage of the ester linkages [3,4,5]. As expected, the new PGS biocomposite scaffolds degraded over time by hydrolysis, hence losing weight. As also reported by Pomerantseva et al. [29], the mass loss rate was constant after the initial period of evaluation and the swelling and water uptake were not significantly detected, due to increased crosslink and density of PGS obtained by the longer curing process during its synthesis. The results are also similar to those presented by Wang et al. [30], where the PGS hydrolysis occurred mostly by surface erosion, preserving the samples’ geometry and with minimal water uptake. The PGS scaffolds reinforced with the bioactive glass fibers presented higher mass loss over time, due to the rapid dissolution of this reactive phase; however, the rather large standard deviation led to a non-statistically significant difference between the groups.

During the degradation tests, pH changes over time were monitored for all samples (Figure 3). The encountered pH values of the medium (ISO degradation solution) in contact with the pure PGS scaffolds were significantly lower after 2 days of incubation (*p* < 0.05), indicating that acidification due to the polymer degradation had occurred. This phenomenon is widely described in literature [3,18,28] and it is linked to ionization of unreacted carboxylic acid groups (–COOH) in PGS and of the carboxylic acid groups formed by hydrolysis of the PGS ester (–COOR) [18,28]. However, for both composites with BGF concentrations of 5 wt % and 10 wt %, this acidification could be neutralized, with pH values remaining around the physiological level, mainly due to the leaching of Ca, Na, K from the fibers. Another reaction responsible for the pH neutralization is that, according to Liang et al. [18], the bioactive glass fibers reacted with the solution, releasing sodium, calcium, and hydroxide ions, which diffused into the PGS matrix and reacted with the carboxylic acid groups forming metallic carboxylates. This pH neutralization is an important factor for improving PGS biointeraction, since it is well established that the acidic degradation products of polyesters lead to an inflammatory response [28,31].

In our experiments using ISO degradation solution, after 5 days the pH slightly increased again, which could be attributed to the fact that the bioactive glass fibers were on the interior of the samples and totally surrounded by the PGS matrix, so that the fiber dissolution rate mostly depended on the previous degradation of the PGS matrix. When PGS suffered hydrolysis, it opened pathways for the interaction of the glass fibers with the solution, leading to an increase in the alkaline ions concentration in the medium, as can be observed in Figure 4.

Through ATR-FTIR analysis, after SBF tests, it was possible to detect that two new peaks at 1545 and 1574 cm^−1^ appeared with the addition of the bioactive glass fibers into PGS. According to Liang et al. [18] and Chen et al. [28], these peaks appear due to the metallic carboxylate stretches of the sodium or calcium carboxylates, which are formed when bioactive glass’ metal oxides interact with PGS pre-polymer carboxylic acid groups. These peaks lose intensity as the time of incubation in SBF solution increase, as these sodium or calcium carboxylate compounds formed between PGS and bioactive glass easily dissolve in the presence of water [18,28].

The FTIR spectra also indicate that the breakdown of the crosslinks in the PGS (the peak forming at 1700 cm^−1^), linked to the polymer’s degradation, starts to occur later for the reinforced samples, mainly for the PGS + 10% BGF composite. This phenomenon is likely related to the formation of these metallic carboxylates groups during fabrication of the biocomposites—they consume PGS carboxylic groups, thus reducing the level of esterification in the PGS matrix. These reactions confirm the improvement of the mechanical and degradation properties of PGS by incorporating BGF, a relevant feature for a biomaterial aimed at cartilage regeneration, since PGS exhibits an accelerated rate of degradation in vivo [3].

The identification of the HCA layer formation, detected by SEM (Figure 7) and XRD analysis (Figure 8), reflected the gain in the biocomposites’ bioactivity with the addition of the glass fibers, potentially increasing the materials biointeraction in vivo. Such composites with ability to form a surface hydroxyapatite layer are interesting for the development of scaffolds for osteochondral regeneration, where the bone-side of the scaffold requires such biomineral growth [32].

The mechanical tests revealed that the average maximum tensile strength values and Young’s modulus increased systematically with the increase of the bioactive glass fiber content in the composite, with a systematic decrease in the strain at break (Figure 9). Tensile stress increased approximately 205% for the PGS + 10% BGF, while the elongation decreased almost 40% when compared to pure PGS scaffolds. This is an expected result, since this is generally observed in composites in which a polymer matrix is reinforced with a rigid ceramic phase, increasing the modulus and/or strength that usually occurs at the expense of elongation at break [19].

One of the reasons for the stiffening and the increase in strength of the PGS matrix by the incorporation of the glass fibers is based on the filler effect, in which the ceramic phase hinders the movement of the polymer chains, reducing the amount of readily extendable material in the specimen [18,19]. This filler effect was also reported by Liang et al. [18], who presented an ultimate tensile strength of 1.53 ± 0.12 MPa when incorporating up to 15 wt % of 45S5 bioactive glass particles into a PGS matrix. As fibers are generally more effective in achieving better mechanical reinforcement than particles [19], the scaffolds developed in this study presented a 2.5 ± 0.8 MPa mean tensile strength with a lower ceramic phase content (10 wt %).

In addition to the filler effect, it is also reported by many authors that the polymer components chemically react with the bioactive glass phase during its synthesis, as observed by infrared spectroscopy [18,26,28]. Chen et al. [28] reported that sebacic carboxylic acid groups react with both the glycerol and alkaline oxides of the bioactive glass, forming ester groups and calcium and sodium dicarboxylate bridges that act as ionic crosslinks, thus increasing PGS strand density, Young’s modulus, and tensile strength of the biocomposites.

These preliminary tests have demonstrated promising results for the application of this novel bioactive composite containing glass fibers as a new biomaterial for soft tissue engineering. Plans for future work include additional in vitro tests to confirm the response of the novel composites in relevant cell lines.

## 4. Materials and Methods

### 4.1. Glass Fiber Manufacture

For manufacturing the bioactive glass fibers, a brand new highly bioactive glass formulation, denominated F18, was used. The manufacture process of this glass is described in detail elsewhere [33,34,35]. Briefly, this new composition belongs to the system SiO_2_–Na_2_O–K_2_O–MgO–CaO–P_2_O_5_, and the glass was prepared by melting analytical-grade chemicals at 1200 °C in a platinum crucible, following by repeated crushing and remelting at 1200 °C to provide homogenization. This glass composition allowed the obtainment of continuous fibers with precise diameter control by the downdrawing process. In our laboratory scale production, 300 g of glass was placed in the furnace and heated above the liquidus temperature (approximately 1250 °C). The viscosity was then adjusted (by tuning the temperature) to be around 10^2^ to 10^3^ Pa·s, and then the glass slowly drained from the Pt crucible’s nozzles. Continuous fibers were pulled mechanically with a controlled velocity for diameter control. The obtained glass fibers had a mean diameter of approximately 20 µm (±5.1 µm).

### 4.2. Fabrication of the PGS-Reinforced Scaffolds

For the PGS synthesis, 14.6 mL of glycerol (Sigma Aldrich, Steinheim, Germany) and 40.4 g of sebacic acid (Sigma Aldrich, Steinheim, Germany) were used. The reagents were stirred at 120 °C for 48 h in a round-bottom three-joint glass flask with a continued nitrogen flux.

The fiber-reinforced PGS scaffolds were prepared by the salt-leaching process with the addition of 0.5 g or 1 g of chopped bioactive glass fibers into 10 g of PGS (PGS + 5 wt % BGF and PGS + 10 wt % BGF, respectively). The fibers had mean length of approximately 1 mm and the mixture was kept stirring for 5 min at 70 °C before casting. Then, the viscous solutions were poured in Teflon plaques of 6 cm of diameter, containing 20 g of NaCl (with a particle size range of 325–500 µm) and placed in a vacuum furnace for 4 days at 140 °C ± 1 °C. After this crosslinking process, discs of 5 mm in thickness were obtained, and the NaCl particles were solubilized in deionized water for 6 h.

### 4.3. Characterization of the Biocomposites

#### 4.3.1. Biocomposite Morphology

Stereomicroscopy (Leica, Wetzlar, Hessen, Germany) and scanning electron microscopy (SEM) (FEG XL30, Philips, Amsterdam, The Netherlands) observations were conducted to evaluate the morphology, interaction and dispersion of the bioactive glass fibers in the PGS matrix.

#### 4.3.2. Degradation Tests

To analyze mass and pH changes over time, degradation tests were performed using a modified ISO standard 10993-14 [20]. A TRIS + HCl solution (ISO degradation solution) was used, and samples were incubated for 2, 7, 14, 21, and 28 days. After the soaking time, the samples were dried at room temperature for 24 h and then weighed with a 0.0001 g accuracy balance (AUW220D, Shimadzu, Kyoto, Japan). All these measurements were conducted in duplicate. After these analyses, ion-selective tests were performed to quantify ion release (Ca, Na, P, and K) over time.

#### 4.3.3. Bioactivity Tests

To evaluate the new composites’ bioactivity, which is related to the formation of hydroxyapatite on the surface of the samples, scaffolds with 10 × 5 × 5 mm^3^ were soaked in 25 mL of SBF-K9 solution, prepared according to the procedure proposed by Kokubo et al. [36], and incubated in vitro at 37 °C for different periods of time (2, 7, and 14 days). After each period, the samples were dried at room temperature for 24 h and subjected to ATR-FTIR spectroscopy over the range of 4000–400 cm^−1^ (Tensor 27, Bruker, MA, USA). SEM images were used to analyze the changes in the glass fibers and in the PGS matrix morphology at the different periods of incubation. X-ray diffraction (XRD) tests (Ultima IV, Rigaku, Tokyo, Japan) were also conducted to identify any crystalline phase precipitated on the samples surface after the immersion in SBF solution.

#### 4.3.4. Mechanical Properties

All composites’ tensile strength was analyzed using a uniaxial testing machine (Zwick, Z050, Ulm, Germany) with a 50 kN load cell at a cross-head speed of 5 mm/min at ambient conditions. All samples were prepared in a prismatic shape with dimensions of 40 mm × 5 mm with a thickness of approximately 2 mm. At least six samples were tested for each type of composite and the average value was reported with standard deviation (±SD) [37].

## 5. Conclusions

In this study, we developed bioactive glass (F18) fiber-reinforced PGS biocomposites. The incorporation of the bioactive phase into the PGS porous matrix allowed the manufacture of a more reactive biocomposite with better mechanical properties and controlled degradation rate when compared with those of the polymer alone. Additionally, the presence of the bioactive fibers could effectively counteract the acidity caused by the degradation of PGS in vitro. These preliminary tests have demonstrated promising results for the application of this novel bioactive composite containing glass fibers as a new biomaterial for soft tissue engineering, for example, cartilage regeneration.

## Figures and Tables

**Table 1:** Mean elongation (%) and tensile strength for pure porous PGS and for the biocomposites PGS + 5% BGF and PGS + 10% BGF.

Material	Mean Strain (%)	Mean Tensile Strength (MPa)
Pure PGS Scaffold	75 ± 14	1.2 ± 0.2
PGS + 5% BG Fibers Scaffold	32 ± 10	1.8 ± 0.5
PGS + 10% BG Fibers Scaffold	30 ± 10	2.5± 0.8
